# Patient‐ and physician‐reported radiation‐induced toxicity of short‐course radiotherapy with a prolonged interval to surgery for rectal cancer

**DOI:** 10.1111/codi.16315

**Published:** 2022-09-20

**Authors:** Maaike E. Verweij, Sieske Hoendervangers, Charlotte M. von Hebel, Apollo Pronk, Anandi H. W. Schiphorst, Esther C. J. Consten, Anke B. Smits, Joost T. Heikens, Emiel G. G. Verdaasdonk, Tom Rozema, Helena M. Verkooijen, Wilhelmina M. U. van Grevenstein, Martijn P. W. Intven

**Affiliations:** ^1^ Division of Imaging and Oncology University Medical Centre Utrecht Utrecht The Netherlands; ^2^ Department of Surgery Jeroen Bosch Hospital Den Bosch The Netherlands; ^3^ Department of Surgery Diakonessenhuis Utrecht The Netherlands; ^4^ Department of Surgery Meander Medical Centre Amersfoort The Netherlands; ^5^ Department of Surgery University Medical Centre Groningen Groningen The Netherlands; ^6^ Department of Surgery St Antonius Hospital Nieuwegein The Netherlands; ^7^ Department of Surgery Rivierenland Hospital Tiel The Netherlands; ^8^ Department of Radiotherapy Verbeeten Institute Tilburg The Netherlands

**Keywords:** low anterior resection syndrome, patient‐reported outcomes, radiation‐induced toxicity, rectal cancer, short course radiotherapy

## Abstract

**Aim:**

A prolonged interval (>4 weeks) between short‐course radiotherapy (25 Gy in five fractions) (SCRT‐delay) and total mesorectal excision for rectal cancer has been associated with a decreased postoperative complication rate and offers the possibility of organ preservation in the case of a complete tumour response. This prospective cohort study systematically evaluated patient‐reported bowel dysfunction and physician‐reported radiation‐induced toxicity for 8 weeks following SCRT‐delay.

**Method:**

Patients who were referred for SCRT‐delay for intermediate risk, oligometastatic or locally advanced rectal cancer were included. Repeated measurements were done for patient‐reported bowel dysfunction (measured by the low anterior resection syndrome [LARS] questionnaire and categorized as no, minor or major LARS) and physician‐reported radiation‐induced toxicity (according to Common Terminology Criteria for Adverse Events version 4.0) before start of treatment (baseline), at completion of SCRT and 1, 2, 3, 4, 6 and 8 weeks thereafter.

**Results:**

Fifty‐one patients were included; 31 (61%) were men and the median age was 67 years (range 44–91). Patient‐reported bowel dysfunction and physician‐reported radiation‐induced toxicity peaked at weeks 1–2 after completion of SCRT and gradually declined thereafter. Major LARS was reported by 44 patients (92%) at some time during SCRT‐delay. Grade 3 radiation‐induced toxicity was reported in 17 patients (33%) and concerned predominantly diarrhoea. No Grade 4–5 radiation‐induced toxicity occurred.

**Conclusion:**

During SCRT‐delay, almost every patient experiences temporary mild–moderate radiation‐induced toxicity and major LARS, but life‐threatening toxicity is rare. SCRT‐delay is a safe alternative to SCRT‐direct surgery that should be proposed when counselling rectal cancer patients on neoadjuvant strategies.

**WHAT DOES THIS PAPER ADD TO THE LITERATURE?**

Information on side effects is needed for counselling rectal cancer patients on neoadjuvant treatment strategies. This paper shows that during short‐course radiotherapy with a prolonged interval to surgery, mild–moderate toxicity is highly prevalent at 1–2 weeks after completion of radiotherapy and gradually declines thereafter. Life‐threatening toxicity is rare.

## INTRODUCTION

Preoperative short‐course radiotherapy (SCRT) (25 Gy in five fractions) and long‐course chemoradiation (CRT) (50 Gy in 25 fractions combined with a radiosensitizer) are two common neoadjuvant regimens for the treatment of rectal cancer [1, 2]. An interval of more than 8 weeks between CRT and total mesorectal excision (TME) for locally advanced rectal cancer has been known to improve tumour downstaging without compromising the postoperative complication rate [3]. In contrast, the recommended interval between SCRT and TME for intermediate risk rectal cancer was less than 1 week (SCRT‐direct surgery), conforming with the treatment schedules of the Swedish Rectal Cancer and Dutch TME trials [4, 5]. SCRT with a prolonged interval to TME (4 weeks or more, SCRT‐delay) was reserved for patients with locally advanced rectal cancer who were too frail to receive CRT [1].

Recently, SCRT‐delay has become a treatment option for a broader range of rectal cancer stages. The randomized Stockholm III trial showed that SCRT‐delay results in a significant reduction of postoperative complications (41% vs. 53%, *P* = 0.001) and an improved pathological complete response rate (10% vs. 0.3%, *P* < 0.001) compared to SCRT‐direct surgery for resectable rectal cancer [6, 7]. The Dutch M1 trial demonstrated that SCRT‐delay and neoadjuvant chemotherapy results in good overall survival (median 3.8 years) for oligometastatic (M1) rectal cancer [8]. Furthermore, the randomized RAPIDO trial showed that SCRT‐delay and neoadjuvant chemotherapy results in decreased disease‐related treatment failure rate (24% vs. 30%, *P* = 0.019) compared to standard CRT and TME in patients with locally advanced rectal cancer [9].

A drawback of SCRT‐delay is the occurrence of radiation‐induced toxicity during the interval. Information on the course of the side effects would be useful for patient counselling on neoadjuvant treatment strategies. This prospective cohort study structurally evaluated patient‐reported bowel dysfunction and physician‐reported radiation‐induced toxicity during the 8 weeks following SCRT‐delay for rectal cancer.

## MATERIALS AND METHODS

### Patients and treatment

Patients were included between December 2018 and June 2021 in the University Medical Centre Utrecht and between July 2020 and June 2021 in the Jeroen Bosch Hospital. Patients were eligible if they were referred for SCRT‐delay (defined as an interval of at least 4 weeks between completion of SCRT and TME) for either intermediate risk rectal cancer (T1–3(distance to the mesorectal fascia >1 mm [MRF−])N1M0 or T3cd(MRF−)N0M0), locally advanced rectal cancer and contraindication for CRT (T3–4(distance to the mesorectal fascia ≤1 mm [MRF+])NxM0 or TxN2M0) or oligometastatic disease (M1) [10]. Exclusion criteria were inadequate command of the Dutch language, severe cognitive disorder or treatment with palliative intent. All patients provided informed consent for the current study and were asked for informed consent for the Dutch Prospective Colorectal Cancer cohort (PLCRC) [11]. PLCRC is a nationwide cohort study wherein data of adult colorectal patients are collected. The medical ethics committee of the University Medical Centre Utrecht approved PLCRC and waived the current study for ethical review. Clinical data were collected from the electronic medical files and within the PLCRC.

Treatment strategy was decided in a multidisciplinary team meeting. SCRT consisted of 25 Gy in five fractions on consecutive working days. Target volumes were the mesorectum, presacral lymph nodes, internal iliac lymph nodes and, in locally advanced rectal cancer, the obturator region [12]. Radiotherapy was administered on either a magnetic resonance guided linear accelerator (MR‐Linac) or a conventional Linac. Planning target volume margins used for the mesorectum and elective lymph node regions were 10 and 8 mm on a conventional accelerator and 4–6 and 4 mm on the MR‐Linac [13]. Treatment was delivered using a volumetric modulated arc therapy technique on the conventional accelerator or an online adapted MRI‐guided intensity modulated radiotherapy technique on the MR‐Linac. Patients with oligometastatic disease received additional treatment (i.e., neoadjuvant chemotherapy and/or liver surgery) after SCRT. Surgery according to the principles of TME was performed at the referral hospitals.

### End‐points

Bowel dysfunction and radiation‐induced toxicity were measured before the start of radiotherapy, at completion of SCRT and at 1, 2, 3, 4, 6 and 8 weeks thereafter. Patients were censored at the time of TME when TME was performed before 8 weeks after completion of SCRT. Bowel dysfunction was measured by the low anterior resection syndrome (LARS) score questionnaire and recorded in a paper or online diary [14]. The LARS score questionnaire consists of five questions on ‘incontinence for flatus’, ‘incontinence for liquid stools’, ‘frequency’, ‘clustering’ and ‘urgency’. These questions add up to a weighted sum that is categorized as no LARS (0–20), minor LARS (21–29) or major LARS (30–42). The LARS score questionnaire and its Dutch translation have been validated for measuring bowel dysfunction after low anterior resection (LAR) [15, 16]. This short questionnaire was used because it is well suited for repeated measurements of bowel function. Radiation‐induced toxicity was recorded during telephone consultations by a physician for diarrhoea, fatigue, cystitis, urinary incontinence and dermatitis according to the Common Terminology Criteria for Adverse Events (CTCAE) version 4.0 [17]. In the case of missing toxicity, the CTCAE score were retrospectively retrieved from the electronic medical files (*n* = 23 at baseline, *n* = 8 at completion of SCRT and *n* = 2 at 1 week after completion of SCRT). Non‐prespecified complaints and additional treatments during SCRT‐delay were retrieved from the electronic medical files and were censored at the start of chemotherapy when chemotherapy was administered within 8 weeks following completion of SCRT.

### Statistical methods

Baseline characteristics were described as number (proportion) or median (range or interquartile range [IQR]). The LARS score questionnaire was processed according to its manual [14]. The LARS score questionnaires and radiation‐induced toxicity measurements were reported as number (proportion) of patients per category or grade per week.

In order to personalize information for future patients about the severity of bowel dysfunction they may expect during SCRT‐delay, the course of LARS was described for several subgroups: neoadjuvant treatment (radiotherapy only vs. radiotherapy and chemotherapy), clinical tumour stage (cT2 vs. cT3 and MRF− vs. cT3 MRF+ and T4), tumour location (distal [lower border of the tumour 0–3 cm from anorectal junction on sagittal MRI] vs. midrectal [3–6 cm] vs. proximal [≥6 cm]), age (40–60 vs. 60–80 vs. 80+ years), gender (male vs. female) and LARS score at baseline (no or minor LARS vs. major LARS).

## RESULTS

Fifty‐one patients including 31 men (61%) were enrolled (Table 1). The median age was 67 years (range 44–91). The indication for SCRT‐delay was intermediate risk rectal cancer in 32 patients (63%), locally advanced rectal cancer and frailty in five patients (10%) and oligometastatic disease in 14 patients (28%). Ten out of 14 patients with oligometastatic disease (71%) were treated with chemotherapy at 14 days after completion of SCRT (median, IQR 12–18) and seven patients with oligometastatic disease (50%) had liver surgery at 157 days (median, IQR 56–180) after completion of SCRT. Of all patients, TME was performed in 40 patients (78%) at 72 days (median, IQR 53–102) after completion of SCRT. Four patients (7.8%) did not undergo TME due to disease progression and three patients (5.9%) declined or were judged unfit to undergo TME (Supplementary File 1). Four patients (7.8%) with a (rectal) clinical complete response entered a watch and wait follow‐up programme.

**TABLE 1:** Patient, tumour and treatment characteristics of 51 rectal cancer patients treated with short‐course radiotherapy and prolonged interval to surgery

	*N* (%)
Male gender	31 (61)
Age in years (median, range)	67 (44, 91)
CCI (%)	
0	33 (65)
1–2	11 (22)
3+	7 (14)
Ostomy before start of treatment	3 (5.9)
Clinical tumour stage	
cT2	9 (18)
cT3	38 (75)
cT4	4 (7.8)
Involvement of mesorectal fascia (≤1 mm, MRF+)	11 (22)
Clinical nodal stage	
cN0	7 (14)
cN1	38 (75)
cN2	7 (14)
Clinical metastasis stage M1	14 (28)
Tumour location^a^	
Distal (0–3 cm)	15 (31)
Midrectal (3–6 cm)	15 (31)
Proximal (6+ cm)	21 (41)
Indication for SCRT	
Intermediate risk rectal cancer^b^	32 (63)
Locally advanced rectal cancer	5 (9.8)
cM1 rectal cancer	14 (28)
Treatment on MR‐Linac	26 (51)
Definitive treatment	
TME	40 (78)
Watch and wait^c^	4 (7.8)
No TME due to distant disease progression	4 (7.8)
No TME due to patient being unfit for surgery	3 (5.9)
Days between completion of SCRT and TME (median, IQR)	72 (53, 102)

Subgroup of cM1 patients (*n* = 14)	
Chemotherapy during interval	10 (71)
Days between completion of SCRT and start of chemotherapy (median, IQR)	14 (12, 18)
Liver surgery	7 (50)
Days between completion of SCRT and liver surgery (median, IQR)	157 (56, 180)

Abbreviations: CCI, Charlson Comorbidity Index (calculated excluding patient age and the rectal tumour); IQR, interquartile range; MR‐Linac, magnetic resonance guided linear accelerator; MRF, mesorectal fascia; SCRT, short‐course radiotherapy; TME, total mesorectal excision.

^a^ Measured as distance between lower border of the tumour and anorectal junction on sagittal MRI.

^b^ Rectal cancer stage 1–3(MRF−)N1M0 or T3c‐d(MRF−)N0M0 according to the Dutch guideline.

^c^ One patient entered watch and wait after a transanal minimal invasive surgical (TAMIS) procedure without residual tumour cells on pathology.

**FIGURE 1:**
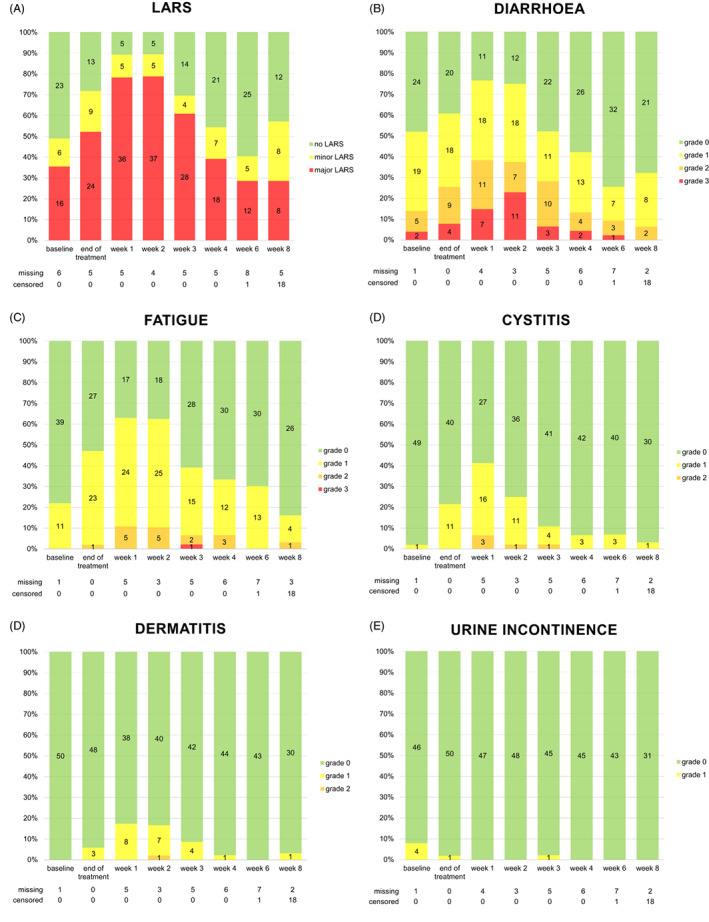
(A)–(F) Patient‐reported bowel dysfunction measured by the low anterior resection syndrome (LARS) score and physician‐reported radiation‐induced toxicity according to CTCAE during short‐course radiotherapy and prolonged interval to surgery (SCRT‐delay) for rectal cancer (*n* = 51). Patients were censored at the time of TME when TME was scheduled within 8 weeks after completion of SCRT.

Both patient‐reported bowel dysfunction and physician‐reported radiation‐induced toxicity peaked at 1–2 weeks after completion of SCRT and gradually declined thereafter (Figure 1; Supplementary File 2). As an exception, the LARS score component ‘incontinence for flatus’ and physician‐reported urine incontinence did not show a clear pattern.

At its peak incidence, major LARS was reported by 37 patients (79%). As for the components of the LARS score questionnaire, clustering of stools that occurred at least once a week was reported by up to 41 patients (85%), urge at least once a week by 37 (79%), incontinence for flatus at least once a week by 26 (57%), defaecation frequency of more than seven times a day by 17 (35%) and incontinence for liquid stools at least once a week by 13 patients (27%). In total, 44 patients (92%) reported major LARS at some time during SCRT‐delay.

At its peak incidence, radiation‐induced diarrhoea was observed in 36 patients (77%), fatigue in 29 (63%), cystitis in 19 (41%), dermatitis in eight (17%) and urine incontinence in four (8.0%). In total, radiation‐induced toxicity Grade 3 diarrhoea occurred in 16 patients (31%) and one (2.0%) had Grade 3 fatigue. In one patient, TME was moved up to 4 weeks due to persisting Grade 3 diarrhoea. No Grade 4–5 radiation‐induced toxicity occurred.

Outside the prespecified toxicities, 42 (82%) patients reported rectal haemorrhage, 21 (41%) rectal or anal pain, 19 (37%) incontinence for solid stools, 17 (33%) abdominal pain, 14 (27%) constipation, 14 (27%) anorexia/nausea, nine (18%) urinary tract obstruction and two (3.8%) neuropathic buttock pain during SCRT‐delay and before the start of chemotherapy (additional treatments during SCRT‐delay are reported in Supplementary File 3).

Subgroup analysis showed that the vast majority of patients treated with neoadjuvant chemotherapy continued to report major LARS at weeks 3 and 4 after completion of SCRT, while the incidence of major LARS already declined in patients treated with radiotherapy only (Supplementary File 4). The majority of patients with cT3MRF+ and cT4 continued to report major LARS throughout follow‐up, and they consistently reported more major LARS than patients with cT3MRF− or cT2. Patients with proximal, midrectal or distal tumours reported similar levels of major LARS. Patients aged 80 years or older consistently reported more major LARS than patients of 60–80 years, who reported more major LARS than patients of 40–60 years. Female patients consistently reported more major LARS than men. Most patients with major LARS at baseline continued to report major LARS throughout follow‐up.

## DISCUSSION

During SCRT‐delay for rectal cancer, patient‐reported major LARS and physician‐reported radiation‐induced toxicity Grades 1–2 were highly prevalent at 1–2 weeks after completion of SCRT and gradually declined thereafter. Radiation‐induced toxicity Grade 3 occurred in total in 33% of patients and consisted predominantly of diarrhoea. No Grade 4–5 radiation‐induced toxicity occurred. Patients treated with neoadjuvant chemotherapy with a higher clinical tumour stage, older age, female gender and major LARS at baseline reported more major LARS during SCRT‐delay. Patients reported more major LARS than physicians reported Grade 3 radiation‐induced diarrhoea.

This is the first study that provides a detailed insight into the course of radiation‐induced toxicity during SCRT‐delay. Previous studies have only reported on cumulative toxicity incidences following SCRT‐delay. A 2014 meta‐analysis by Bujko et al. reported that radiation‐induced toxicity occurred in 27%–41% of patients during SCRT‐delay, of whom 2%–5% had Grade ≥3 toxicity [18]. In the Stockholm III trial, 7% (*n* = 23/355) of patients treated with SCRT‐delay were admitted to the hospital due to radiation‐induced toxicity [6]. In our study, no Grade 4–5 toxicity occurred and in only one patient TME was moved up due to persisting Grade 3 toxicity. Advances in radiotherapy techniques since the start of the Stockholm III trial, that included patients between 1998 and 2013, might explain the lower toxicity rates in our cohort. In contrast, no Grade 3–4 radiation‐induced toxicity occurred during SCRT‐delay and before the start of chemotherapy in the M1 trial. Administration of chemotherapy was delayed in seven (14%) patients due to Grade 2 radiation toxicity at 2 weeks after completion of SCRT [19]. The relatively favourable toxicity results of the M1 trial might be explained by their young and fit study population (median age 59 [range 33–75] and Eastern Cooperative Oncology Group 0 or 1). Our study shows that, using current radiotherapy techniques in an all‐comer population, almost every patient experiences temporary mild–moderate radiation‐induced toxicity during SCRT‐delay, but life‐threatening radiation‐induced toxicity is rare. Combining our results with the lower risk of postoperative complications and the increased probability of organ preservation, SCRT‐delay should be preferred over SCRT‐direct surgery in most rectal cancer patients [6, 7]. SCRT‐direct surgery could still be considered for patients with no interest in organ preservation and/or a high risk of radiation‐induced toxicity following SCRT‐delay.

Neoadjuvant chemotherapy, higher clinical tumour stage, older age, female gender and major LARS at baseline were associated with major LARS during SCRT‐delay. Previous studies found a distal tumour, female gender and a younger age to be predictive of the LARS score at one or more years after anterior resection [14, 20]. However, the relation between younger age and bowel dysfunction might have been biased by the selection of patients for anterior resection. Anorectal function decreases with age and is worse in women than in men, especially after (vaginal) childbirth [21, 22]. It is therefore plausible that older and female patients are more susceptible to major LARS following radiotherapy for rectal cancer. Tumours of a higher stage or at a more distal location exert more pressure on the anorectal complex and on the rectal ampulla, so more major LARS was expected in those subgroups. A high clinical tumour stage was strongly associated with the occurrence of major LARS, but tumour location was not associated with LARS. Chemotherapy was associated with a slower recovery of LARS after SCRT. These risk factors should be considered when counselling patients on LARS during SCRT‐delay.

Patient‐reported LARS and physician‐reported radiation‐induced diarrhoea showed similar patterns, but a considerable proportion of patients reported major LARS when physicians reported diarrhoea Grade 0, 1 or 2. This difference is probably due to the extensiveness of the LARS score questionnaire compared to diarrhoea according to the CTCAE grading system [14]. When interpreting the LARS score, it should be acknowledged that major LARS has a prevalence of 15% in a reference population [23, 24]. Also, it is well known that physicians consistently report lower frequency and severity of toxicity than patients do in direct reports [25]. Our study once again shows the importance of collecting patient‐reported outcomes for measuring the impact of a treatment.

The LARS score questionnaire has been validated for measuring bowel dysfunction after LAR [14, 15, 16, 26]. Here, the LARS score questionnaire was used to measure bowel dysfunction following radiotherapy for rectal cancer, an indication for which it has not been specifically validated. That the LARS score questionnaire does not cover all radiation‐induced bowel symptoms is illustrated by the high prevalence of rectal haemorrhage (82%), rectal pain (41%) and incontinence for solid stools (37%) in our study. However, major LARS has been correlated with poor quality of life in a reference population, indicating that the LARS score questionnaire is of value outside of the LAR population [23]. Other studies have used the LARS score questionnaire in patients who had not been treated with LAR, that is, patients on a watch‐and‐wait strategy [27, 28, 29]. Future research could focus on the development and validation of a simple questionnaire like the LARS score questionnaire for measuring bowel dysfunction following radio(chemo)therapy for rectal cancer.

In this study, toxicity was only recorded for diarrhoea, fatigue, cystitis, urine incontinence and dermatitis during 8 weeks following completion of SCRT. Adverse events during the remaining duration of chemotherapy were not recorded. Because of these choices, it was unfortunately not possible to compare our results to trials that reported the cumulative incidence of toxicity of SCRT‐delay and neoadjuvant chemotherapy together (such as the RAPIDO trial).

Missing values for physician‐reported radiation toxicity and non‐prespecified complaints were retrospectively retrieved from the electrical medical files. Their prevalence might be underestimated due to underreporting.

## CONCLUSION

During SCRT‐delay, almost every patient experiences temporary mild–moderate radiation‐induced toxicity and major LARS, but life‐threatening toxicity is rare. Neoadjuvant chemotherapy, higher clinical tumour stage, older age, female gender and major LARS are risk factors for major LARS during SCRT‐delay. SCRT‐delay is a safe alternative to SCRT‐direct surgery that should be proposed when counselling rectal cancer patients on neoadjuvant treatment strategies.

## AUTHOR CONTRIBUTIONS

Maaike E. Verweij: Investigation, Writing ‐ original draft, Methodology, Validation, Visualization, Software, Formal analysis, Project administration, Data curation. Sieske Hoendervangers: Conceptualization, Investigation, Methodology, Validation, Writing ‐ review & editing, Software, Project administration, Data curation, Visualization. Charlotte M. von Hebel: Investigation, Visualization, Data curation, Software, Validation. Apollo Pronk: Resources. Anandi H. W. Schiphorst: Resources. Esther C. J. Consten: Resources. Anke B. Smits: Resources: Joost T. Heikens: Resources. Emiel G. G. Verdaasdonk: Resources.Tom Rozema: Resources. Helena M. Verkooijen: Conceptualization, Supervision, Writing ‐ review & editing, Methodology, Funding acquisition. Wilhelmina M. U. van Grevenstein: Conceptualization, Funding acquisition, Resources, Supervision, Writing ‐ review & editing, Methodology. Martijn P. W. Intven: Funding acquisition, Conceptualization, Resources, Supervision, Writing ‐ review & editing, Methodology.

## FUNDING INFORMATION

No funding has been received by any author in relation to this article.

## CONFLICT OF INTEREST

Outside of the submitted work, HMV is a member of the European Commission and the Netherlands Organization of Health Research and Development and reports grants for Elekta AB, Sweden and the Dutch Cancer Foundation. MPWI has received personal fees from Elekta AB, Sweden.

## ETHICAL APPROVAL

The medical ethics committee of the University Medical Centre Utrecht waived the current study for ethical review.

## PATIENT CONSENT STATEMENT

All patients provided informed consent before study inclusion.

## Supporting information


Appendix S1

## Data Availability

The data that support the findings of this study are available from the corresponding author upon reasonable request.
