# Supplementary material for: Patient‐ and physician‐reported radiation‐induced toxicity of short‐course radiotherapy with a prolonged interval to surgery for rectal cancer
Source: Colorectal Dis. 2022 Sep 20;25(1):24–30. doi: 10.1111/codi.16315 (PMC10087149; doi:10.1111/codi.16315)
Supplement: Supplementary file 1 — Appendix S1 [file CODI-25-24-s001.docx]

**SUPPLEMENTARY FILES**

**Supplementary File 1.** Type of definitive treatment per rectal cancer risk category among 51 rectal cancer patients treated with short course radiotherapy and prolonged interval to surgery.

|  | **Intermediate risk (n=32)** | **Locally advanced (c=5)** | **cM1 (n=14)** |
| --- | --- | --- | --- |
| **TME** | 28 (88) | 2 (40) | 10 (71) |
| **Watch & wait** | 2 (6.3) | 0 | 2 (14.3) |
| **No TME due to distant disease progression** | 1 (3.1) | 1 (20) | 2 (14.3) |
| **No TME due to patient being unfit for surgery** | 1 (3.1) | 2 (40) | 0 |

cM1: oligometastatic rectal cancer. TME: Total mesorectal excision.

**Supplementary File 2.** Number (proportion) of patients reporting each component of the low anterior resection syndrome (LARS) score during short course radiotherapy and prolonged interval to surgery (SCRT-delay) for rectal cancer (n=51). Patients were censored at time of TME when TME was scheduled within 8 weeks after completion of SCRT.


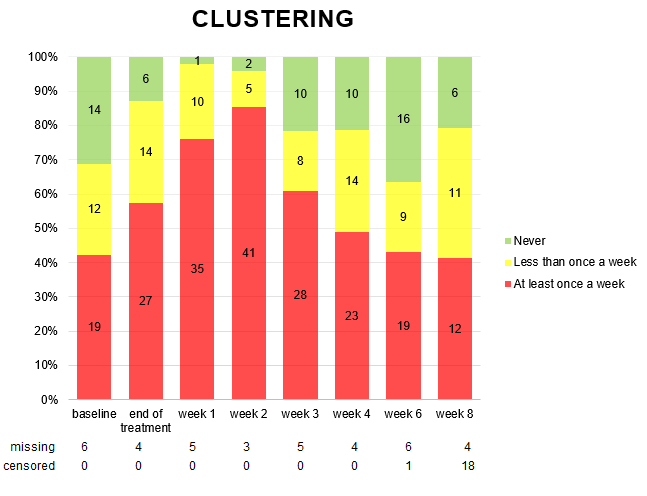


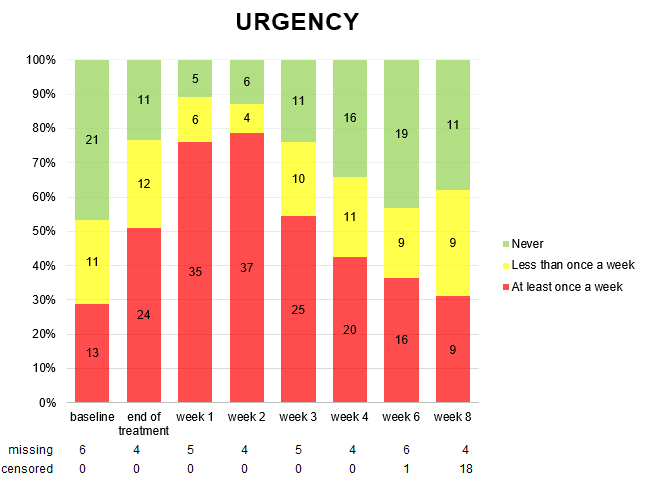


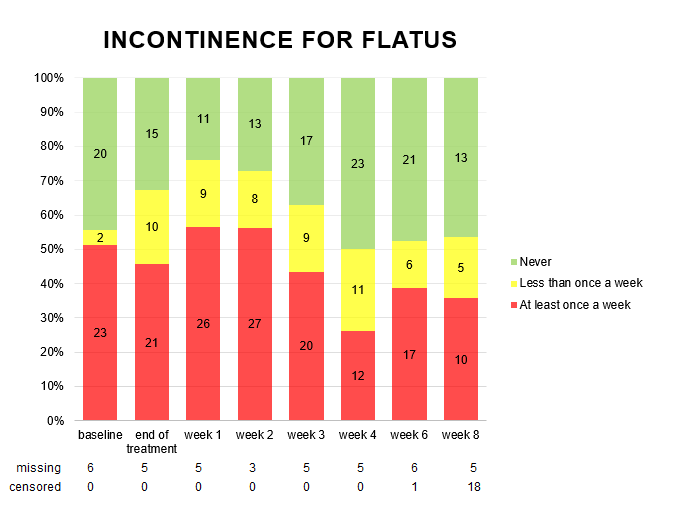


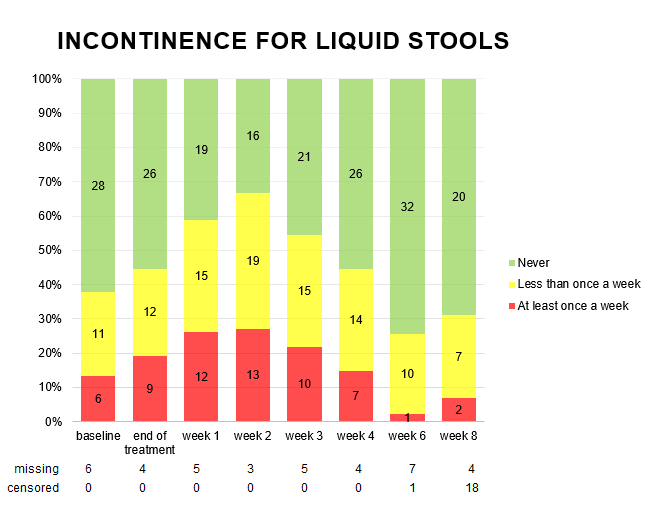


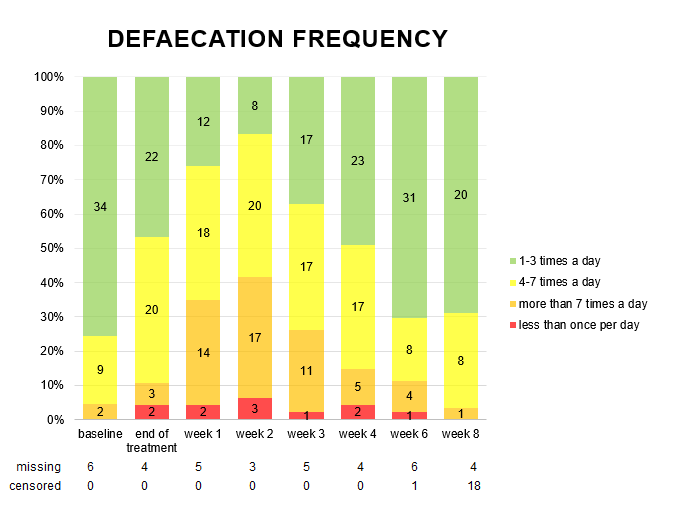


**Clustering**: do you ever have to open your bowels again within one hour of the last bowel opening? **Urgency:** do you ever have such a strong urge to open your bowels that you have to rush to the toilet? **Incontinence for flatus**: do you ever have occasions when you cannot control your flatus (wind)? **Incontinence for liquid stool**: do you ever have any accidental leakage of liquid stool? **Defaecation frequency**: how often do you open your bowels?

**Supplementary File 3.** Additional treatments received during short course radiotherapy and prolonged interval to surgery in 51 patients with rectal cancer.

| **Treatment** | **N (%)** |
| --- | --- |
| Laxatives | 21 (41) |
| Simple analgetics* | 16 (31) |
| Cetomacrogol | 12 (24) |
| Incontinence pads | 9 (18) |
| Loperamide | 8 (16) |
| Morphine based analgetics | 5 (10) |
| Protein rich drinking food | 3 (6) |
| Tamsulosin | 2 (4) |
| Iron suppletion | 1 (2) |
| Blood transfusion | 1 (2) |

*Paracetamol, NSAID’s.

**Supplementary File 4.** Low anterior resection syndrome (LARS) scores during short course radiotherapy and prolonged interval to surgery (SCRT-delay) for rectal cancer in subgroups according to neoadjuvant treatment (radiotherapy only (n=41) vs. chemotherapy during the interval (n=10)), clinical tumour stage (cT2 (n=9) vs. cT3 MRF- vs. cT3 MRF+ (n=31) and cT4(n=11)), tumour location (distal (0-3cm from anorectal junction, n=15) vs. midrectal (3-6cm, n=15) vs. proximal (≥6cm, n=21)), patient age (40-60 (n=15) vs. 60-80 (n=24) vs. 80+ years (n=12)) and LARS complaints at baseline (no or minor LARS (n=29) vs. major LARS (n=16)).


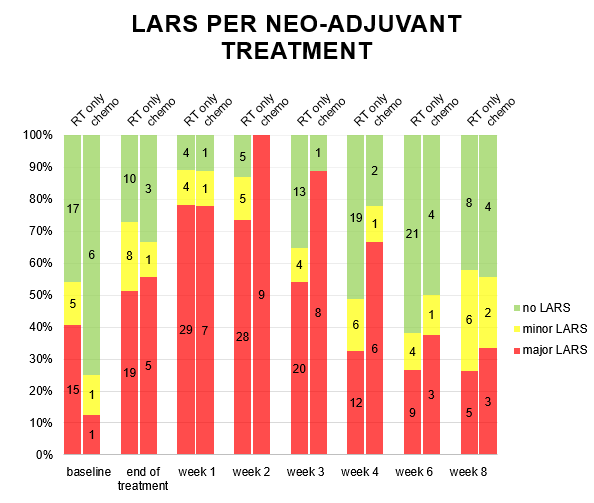


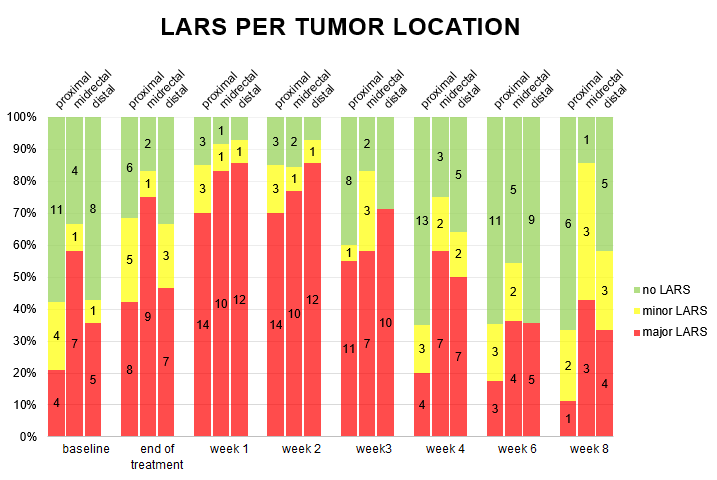

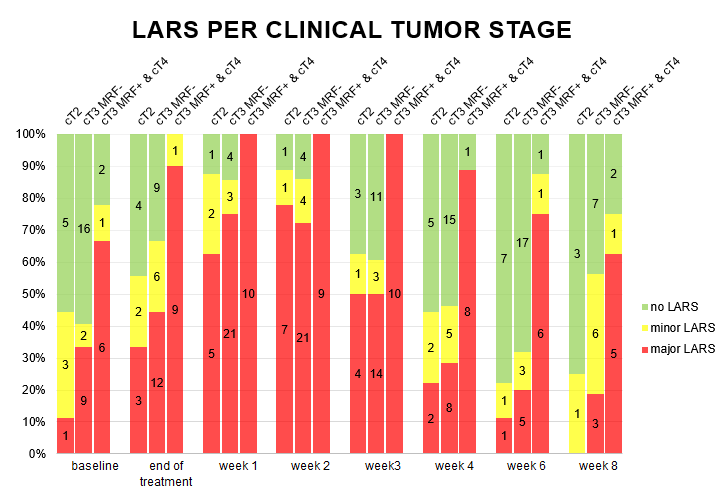


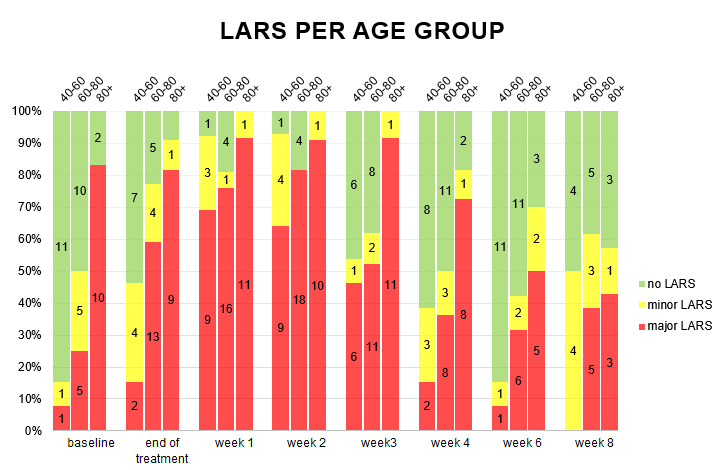
**
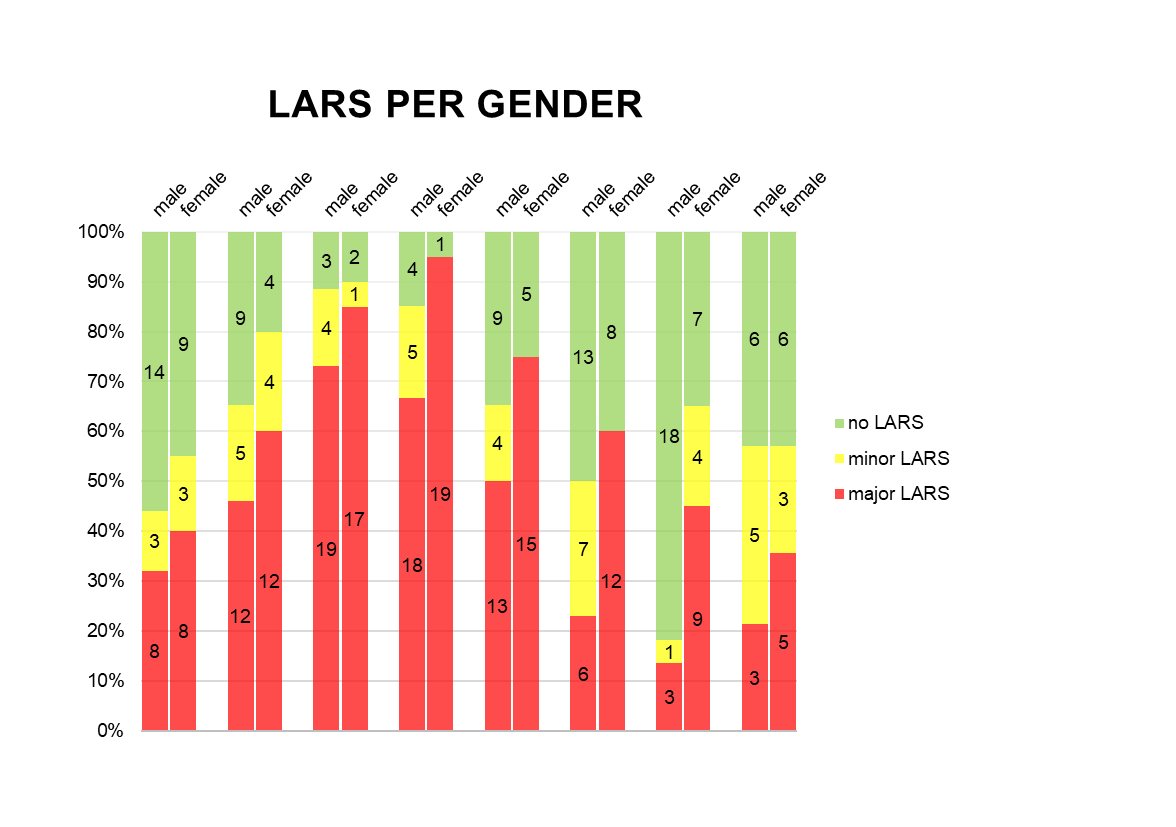
**

**
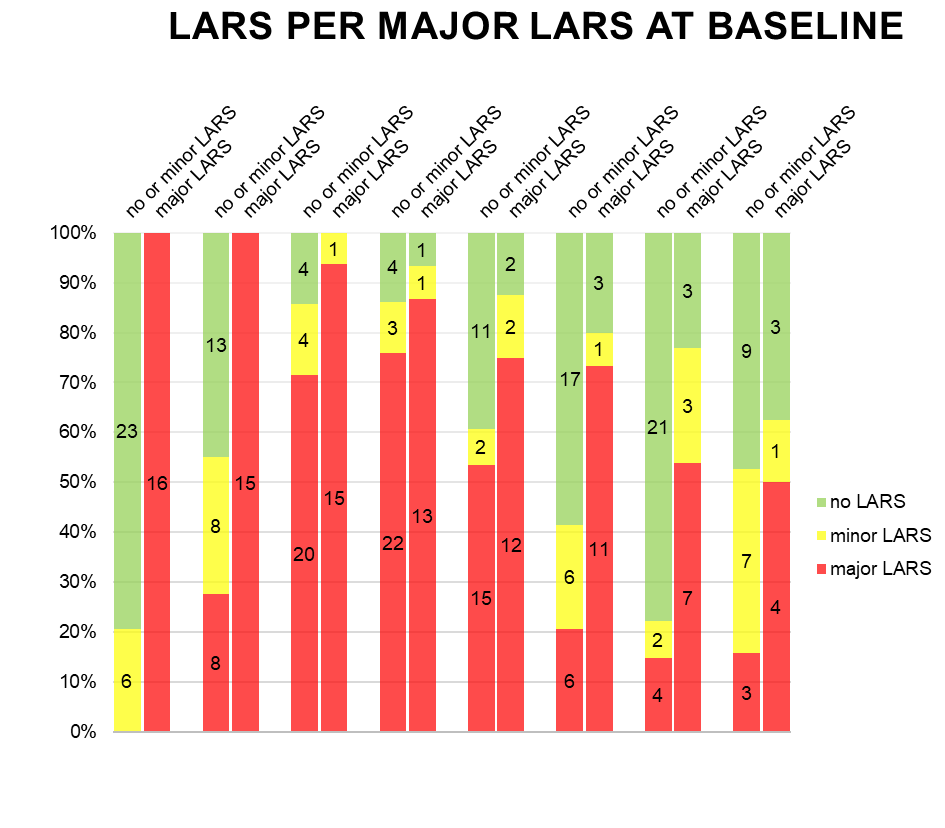
**
